# Increase in Growth Cone Size Correlates with Decrease in Neurite Growth Rate

**DOI:** 10.1155/2016/3497901

**Published:** 2016-05-04

**Authors:** Yuan Ren, Daniel M. Suter

**Affiliations:** ^1^Department of Biological Sciences, Purdue University, West Lafayette, IN 47907, USA; ^2^Bindley Bioscience Center, Purdue University, West Lafayette, IN 47907, USA; ^3^Purdue Institute for Integrative Neuroscience, Purdue University, West Lafayette, IN 47907, USA

## Abstract

Several important discoveries in growth cone cell biology were made possible by the use of growth cones derived from cultured* Aplysia* bag cell neurons, including the characterization of the organization and dynamics of the cytoskeleton. The majority of these* Aplysia* studies focused on large growth cones induced by poly-L-lysine substrates at early stages in cell culture. Under these conditions, the growth cones are in a steady state with very little net advancement. Here, we offer a comprehensive cellular analysis of the motile behavior of* Aplysia* growth cones in culture beyond this pausing state. We found that average growth cone size decreased with cell culture time whereas average growth rate increased. This inverse correlation of growth rate and growth cone size was due to the occurrence of large growth cones with a peripheral domain larger than 100 *μ*m^2^. The large pausing growth cones had central domains that were less consistently aligned with the direction of growth and could be converted into smaller, faster-growing growth cones by addition of a three-dimensional collagen gel. We conclude that the significant lateral expansion of lamellipodia and filopodia as observed during these culture conditions has a negative effect on neurite growth.

## 1. Introduction

The neuronal growth cone is the motile tip of the neurite and an important mediator of neural plasticity [1–3]. It directs axons and dendrites to their respective targets during neural development and regeneration, by sensing and responding to a multitude of both chemical and physical cues [4, 5]. As a result of the extreme sensitivity of growth cones to their environment, dramatic variation in morphology and motility of the same growth cone has often been observed* in vivo*, reflecting the complex nature of the microenvironment [6–8]. Cell culture provides an alternative to study the behavior of growth cones in a defined experimental setting [9–11]. Cultured neurons retain the ability to form neurites as well as axons and dendrites [9, 10, 12, 13]. The growth cones formed* in vitro* are characterized by three domains: (1) the peripheral (P) domain, which includes both filopodial and lamellipodial extensions at the distal edge; (2) the central (C) domain, which is rich in organelles and vesicles and bridges the P domain of growth cone and the newly formed neurite shaft; and (3) the transition (T) zone between P and C domain, often bearing membrane ruffles [1, 2, 5]. This domain assignment is a general feature of growth cones, whereas the relative size and shape of individual domains for a given growth cone vary greatly depending on species, cell type, and culture condition. In a complete cycle of neurite outgrowth, the protrusion of filopodia and lamellipodia in the P domain of growth cone is followed by the invasion of microtubule bundles, organelles, and vesicles into the P domain (engorgement) and the conversion of the growth cone neck into a new segment of the neurite (consolidation) [5, 14–16].

The bag cell neuron from the sea slug* Aplysia californica* is one of the most extensively used model systems for investigations involving electrophysiology [17–19], neuropeptide synthesis and secretion [17, 20–22], neuronal motility and guidance [23, 24], cytoskeletal dynamics [25–29], and cellular biophysics [30–32]. The preference for* Aplysia* bag cell neurons results in part from the large cell body (~50 *μ*m diameter) and the large growth cone in cell culture, especially when plated on poly-L-lysine- (PLL-) coated coverslips (~1000 *μ*m^2^ total growth cone surface area). Comparatively, vertebrate neuronal growth cones are typically smaller than 100 *μ*m^2^. This 10x increase in size is advantageous not only for quantitative high-resolution analysis of numerous growth cone activities [27, 28, 33, 34], but also for various biophysical manipulations [29, 30]. Not surprisingly, several key findings regarding growth cone morphology and dynamics were made in studies using* Aplysia* bag cell neuronal growth cones, thanks to the advantages provided by both the large size and the stereotypic domain organization [23–25, 30, 35]. In addition, the organized geometry of the* Aplysia* growth cone is an attractive target for modeling of growth cone dynamics and motility [36]. Despite the pivotal role that* Aplysia* bag cell neuronal growth cone played in cellular neurobiology, a basic description of growth cone behavior* in vivo* has been missing due to challenges in breeding and imaging developing* Aplysia* [37, 38].

In previous* in vitro* studies involving* Aplysia* bag cell neurons, no staging of growth cone development has been performed as it has been done for hippocampal neurons [10] or* Aplysia* buccal ganglion cell neurons developing normally [14] or after axotomy [39]. Instead, almost all previous studies involving* Aplysia* bag cell growth cones have focused on large growth cones on PLL substrates and short-term motility at steady state, during which neither significant growth nor retraction occurred. Accounts of bag cell growth cones showing significant advancement are rare, and the growth rate was usually lower than that of other types of growth cones, unless neurons were stimulated by apCAM substrates [24, 30, 33, 34] or plated on hemolymph with or without laminin [31, 40]. It is unclear whether the pausing state of large growth cones is due to intrinsic properties or due to limiting factors presented by the culture environment or both.

In this study, we provide a detailed analysis of the behavior of* Aplysia* bag cell growth cones beyond the large, pausing state typically observed in culture. We show that average size of growth cone decreased with increasing cell culture time, whereas average growth rate increased. We found that this inverse correlation was due to the occurrence of large growth cones (with P domains larger than 100 *μ*m^2^) at early stages of cell culture and that large growth cones usually had minimal growth rate (less than 2 *μ*m/h). C domains of large growth cones were less aligned with the direction of growth when compared to C domains of smaller, fast-growing cones. Large growth cones were formed on two-dimensional (2D) PLL substrates by lamellipodial veils connecting numerous filopodia. Furthermore, we found that large growth cones could exit the pausing state either by branching or by application of a type I collagen gel on top of the growth cone. These findings suggest that widely spread growth cones as found on 2D PLL substrates are not conducive to fast neurite growth.

## 2. Materials and Methods

### 2.1. Cell Culture

Bag cell neurons from adult* Aplysia californica* (200 g, Marinus Scientific, Long Beach, CA) were harvested as described previously [11]. Cells were cultured on acid-cleaned coverslip or glass-bottom dishes (MatTek Corporation, Ashland, MA) coated with 20 *μ*g/mL PLL (70–150 kD; Sigma, St. Louis, MO). L15 (Invitrogen; Life Technologies, Grand Island, NY) supplemented with artificial sea water (ASW) was used as culture media (L15-ASW: L15 plus 400 mM NaCl, 9 mM CaCl_2_, 27 mM MgSO_4_, 28 mM MgCl_2_, 4 mM L-glutamine, 50 *μ*g/mL gentamicin, and 5 mM HEPES, pH 7.9, osmolarity 950–1000). Cells were kept at 14°C except for when imaged on the microscope.

### 2.2. Imaging

Live cell imaging was performed at room temperature in L15-ASW on a Nikon TE2000 E2 inverted microscope (Nikon, Melville, NY) with 60x oil immersion objective lens, with additional 1.5x magnification. Focus during long time-lapse sequences was maintained with the Nikon Perfect Focus system. Images were collected with a Cascade II charge-coupled device camera (Photometrics, Tucson, AZ) controlled by MetaMorph 7.8 software (Molecular Devices, Sunnyvale, CA). Imaging chamber was assembled as previously reported for cells cultured on coverslip [41], and fresh L15-ASW was supplemented for imaging longer than 1 h. For cells cultured on glass-bottom dish, fresh medium was exchanged every hour. Time-lapse series were acquired at 30 s intervals for 3.5 h (Figure 4(a)); 60 s intervals for 3 h (Figure 4(b)); 120 s intervals for 13 h (Figure 5(a)); and 30 s intervals for 3 h (Figure 6(g)).

### 2.3. Neurite Growth Rate and Growth Cone Size Analysis

Neurites were identified and imaged at different time points after cell plating: between 24 and 27 h; between 48 and 51 h; and between 72 and 75 h after cell plating. MetaMorph 7.8 software was used for image analysis. To determine individual neurite growth rates, the displacement of a neurite tip over a three-hour interval (3.5 h for Figure 2) was measured and divided by the time. P domain size was measured and used to correlate with the growth rate of an individual growth cone. To account for different shape and domain organization of big and small growth cones, only the P domain directly in front of C domain was included. Specifically, the center of the C domain was determined, which served as the origin of a Cartesian coordinate system superimposed on the growth cone with the *y*-axis in the direction of growth cone advance. Only the area of the P domain that was in front of the *x*-axis was included in the size measurements.

### 2.4. 2D-3D Transition Culture

Cells were cultured on PLL-coated coverslips for 24 h to allow for the formation of growth cones, and then an imaging chamber was assembled. Liquid 2 mg/mL type I rat tail collagen (Corning, Corning, NY) based gel mixture (containing 25%* Aplysia* hemolymph [40] and 20 *μ*g/mL PLL) was introduced into the chamber on top of the cells and allowed to solidify at 37°C for 1 h. The imaging chamber was placed back into dishes with L15-ASW for continued cell culture at RT, until chambers were mounted for imaging. Cells cultured in this setup maintain a healthy morphology for at least 10 d.

### 2.5. Statistics

**Figure 1:**
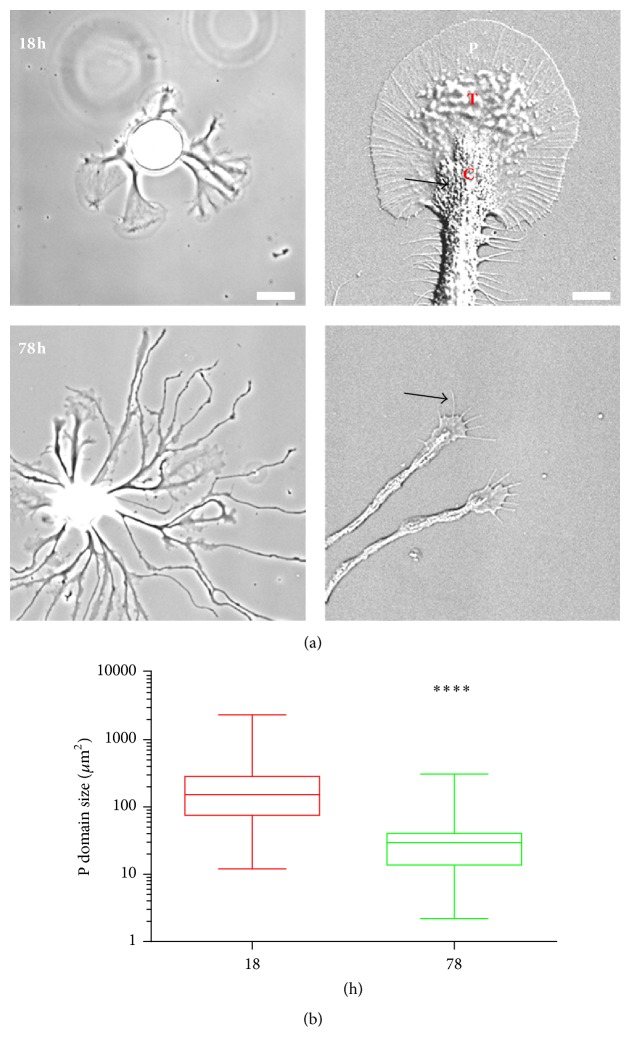
Growth cone size decreases between 18 and 78 h in cell culture. (a) Representative differential interference contrast (DIC) images of* Aplysia* bag cell neurons at 18 h (upper panels) and 78 h (lower panels) after cell plating. Left panels show lower magnification and right panels show higher magnification. P and C domain and T zone are indicated in the upper right image. Arrows point to vesicles in the C domain in the upper right image and to filopodium in the lower right image. (b) Statistical comparison of P domain sizes between 18 h and 78 h, shown in log scale. Average P domain size ± SEM at 18 h is 242.1 ± 39.1 *μ*m^2^ (*n* = 74); average P domain size at 78 h is 37.7 ± 7.5 *μ*m^2^ (*n* = 45). Data are pooled from 3 independent experiments. Box: 25th and 75th percentile plus median line; whisker: min and max. ^*∗∗∗∗*^ *P* < 0.0001. Mann-Whitney test. Scale bar on the left: 60 *μ*m; scale bar on the right: 10 *μ*m.

Graphs were made in GraphPad Prism 6 (GraphPad Software, Inc., La Jolla, CA). Mann-Whitney test (Figures 1 and 6) or Dunn's test (Figure 3) was used following normality testing to compare the P domain sizes and growth rates of different groups. Since P domain and growth cone size data was not normally distributed and varied over a wide range, we used a log scale and whisker plots with the box representing the 25th and 75th percentile, middle line indicating the median, and whiskers representing the min and max values. Significance was established at *P* < 0.05.

## 3. Results

### 3.1. *Aplysia* Growth Cones Become Smaller with Time in Culture

Previous studies on* Aplysia* bag cell neuronal growth cones have mainly focused on large fan-shaped specimens cultured for one or two days on PLL substrates [24, 27, 28, 33, 34]. Such growth cones exhibit not only distinct organization of cytoplasmic domains but also a highly organized actin and microtubule cytoskeleton with well-characterized dynamic properties. However, the vast majority of these growth cones do not significantly translocate during a typical observation time of 30 to 60 min. Here, we compared the sizes of growth cones from cultured bag cell neuron on PLL-coated coverslips at different times in culture. At 18 h after plating, multiple large growth cones with a diameter between 50 and 100 *μ*m appeared around the cell body. These growth cones share stereotypic organization, with a flat P domain and organelle-rich C domain separated by the T zone that features extensive membrane ruffles (Figure 1(a)). By 78 h, growth cones moved further away from the cell body and were considerably smaller in size. The smaller growth cones had fewer filopodia and a less obvious T zone (Figure 1(a)). The portion of the filopodia extending beyond the leading edge became proportionally longer compared to the portion of the filopodia embedded in the lamellipodia (arrow in the lower right image). Statistical analysis revealed a wide distribution of P domain size at both time points (note the log scale), as well as a significant drop of the average P domain size at 78 h when compared to the 18 h time point (Figure 1(b)).

### 3.2. Large Growth Cones Tend to Have Lower Growth Rates

**Figure 2:**
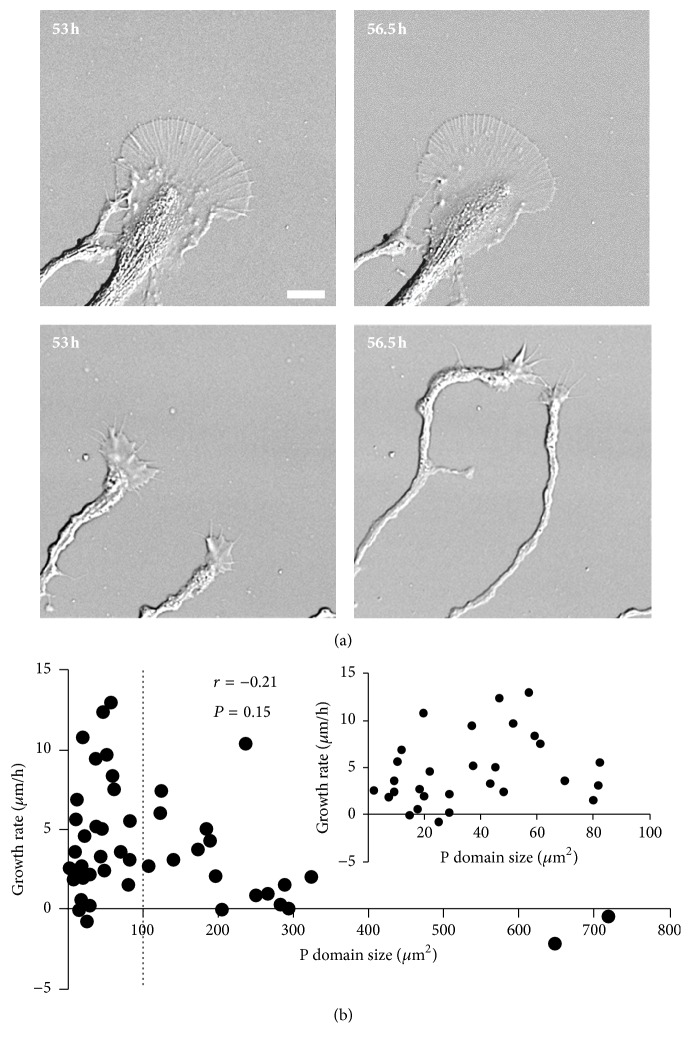
Correlation between P domain size and growth rate. (a) DIC images of examples of a large growth cone (upper panels) and two small growth cones (lower panels) imaged at 53 h (left) and 56.5 h (right), respectively. (b) Plot of growth rate against P domain size for individual growth cones (*n* = 47). Spearman correlation coefficient and *P* value for all growth cones included are shown. Inset shows the data for growth cones with P domain smaller than 100 *μ*m^2^. Data is representative of two independent experiments. Scale bar: 10 *μ*m.

In order to test if growth cone size has an impact on growth rate, we took images of individual growth cones on the second day of plating, at 3.5 h interval (at 53 h and 56.5 h after plating). As can be seen from Figure 2(a), the large growth cone exhibited minimal advancement within 3.5 h, while the two smaller growth cones translocated 45 and 50 *μ*m, respectively, over the same period of time. A plot of growth cone advance rate versus P domain size indicated a nonlinear correlation, with the highest growth rate at around 50 *μ*m^2^ and a decline of growth rate for growth cones with P domain larger than 100 *μ*m^2^ (Figure 2(b)). Growth cones with P domains smaller than 100 *μ*m^2^ did not show a clear correlation between size and advance rate (inset in Figure 2(b)). Growth cones with P domains larger than 200 *μ*m^2^ were less abundant and usually had growth rates of less than 2 *μ*m/h. In conclusion, our results show that large growth cones tend to grow slowly, whereas small growth cones do not have a clear correlation between size and advance rate.

### 3.3. Neurite Growth Rate Increases with Time in Culture as Growth Cone Size Decreases

**Figure 3:**
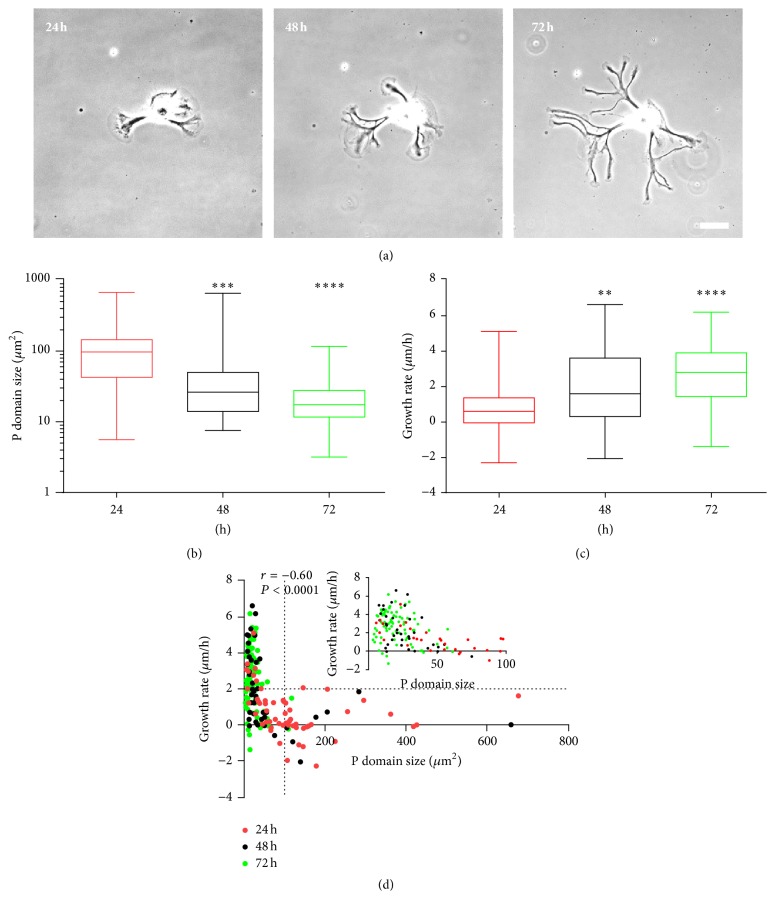
Neurite growth rates increase with decreasing growth cone size and time in culture. (a) A cultured bag cell neuron was imaged at 3 time points after plating (24 h, 48 h, and 72 h). At each time point, the displacement of growth cones over a 3 h time interval was used to calculate the growth rate. (b) Statistical comparison of P domain sizes at 24 h, 48 h, and 72 h after plating, respectively. Average P domain size ± SEM for 24 h is 120.4 ± 16.2 *μ*m^2^, for 48 h is 61.0 ± 16.8 *μ*m^2^, and for 72 h is 22.8 ± 2.2 *μ*m^2^. (c) Statistical comparison of growth rates. Average growth rate ± SEM for 24 h is 0.8 ± 0.2 *μ*m/h, for 48 h is 1.9 ± 0.3 *μ*m/h, and for 72 h is 2.6 ± 0.2 *μ*m/h. (d) Plot of growth rate and P domain size for individual growth cones at different time points. Growth cones from different time points are shown in different color (red: 24 h, *n* = 56; black: 48 h, *n* = 43; green: 72 h, *n* = 80). Spearman correlation coefficient and *P* value are shown for data pooled from three time points. Inset shows the plot only for growth cones with P domain smaller than 100 *μ*m^2^. Plot is representative of two independent experiments. Box: 25th and 75th percentile plus median line; whisker: min and max. ^*∗∗*^ *P* < 0.01; ^*∗∗∗*^ *P* < 0.001; ^*∗∗∗∗*^ *P* < 0.0001. Dunn's test. Scale bar: 50 *μ*m.

Prompted by the findings that growth cone size decreases with increased cell culture time and that larger growth cones exhibit lower growth rates, we set out to test whether an increase in growth rate can be observed at later time points of cell culture. To this end, images of individual growth cones were taken at 3 h intervals at 24, 48, and 72 h after cell plating (Figure 3(a)). As predicted, we observed a significant decrease in average P domain size, concomitant with a significant increase in average growth rate with time in culture (Figures 3(b) and 3(c)). Plots of growth rate versus P domain size for individual growth cones, however, showed a nonlinear correlation (Figure 3(d)) in agreement with data shown in Figure 2(b). These plots revealed that the P domain size is a poor predictor of growth rate for individual growth cones with P domain size smaller than 50 *μ*m^2^; however, it becomes a better predictor for larger growth cones, since 100% of growth cones with P domain size larger than 100 *μ*m^2^ have small growth rate (less than 2 *μ*m/h). In summary, the decreased percentage of large growth cones at later time points of cell culture accounts for the increase in average neurite growth rate with time in culture. At later times in culture, the majority of neurite outgrowth was carried out by smaller growth cones with P domain size of less than 50 *μ*m^2^.

### 3.4. Large and Small Growth Cones Show Different C Domain and T Zone Dynamics

**Figure 4:**
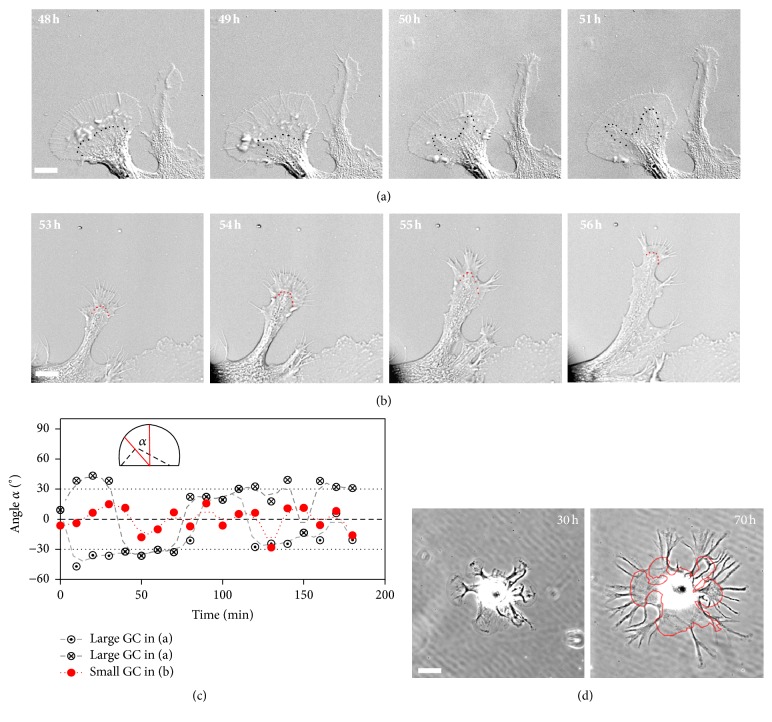
Large and small growth cones show different motile behavior. (a) DIC time-lapse imaging of a large, fan-shaped growth cone on the left revealed minimal net advancement during 3 h, whereas the small growth cone on the right translocated over a distance of 9 *μ*m during the same time period. The C domain boundary is marked with a black dotted line. Note the splitting of C domain at the 50 h time point. (b) DIC time series of a small growth cone that showed significant translocation within 3 h. P domain protrusion is followed by advancement of the C domain (boundary marked with red dotted line). (c) Angle *α* between the direction of the C domain and the direction of growth cone advancement, measured at 10 min intervals. A diagram of C domain with −45 degrees relative to the direction of growth cone advance is shown as an inset. The small growth cone shown in (b) exhibited little fluctuation of the angle, whereas the large growth cone shown in (a) showed swaying of the C domain manifested by larger fluctuations of the angle (crossed circles) or splitting of the C domain (crossed and dotted circles). (d) Images of the same cultured bag cell neuron taken 40 h apart. Outline of growth cones at 30 h is overlaid to the image taken at 70 h. Note the formation of new neurites by branching from edges of P domain. Scale bars in (a) and (b): 10 *μ*m; scale bar in (d): 60 *μ*m.

In order to identify other differences between large and small growth cones besides size that might explain their different growth rates, we used high-resolution live cell imaging over the time course of 3 h to study their motile behavior. In agreement with previous findings (Figure 2), growth cones with P domains larger than 100 *μ*m^2^ rarely showed net advancement (Figure 4(a); Video 1 in Supplementary Material available online at http://dx.doi.org/10.1155/2016/3497901). These large, fan-shaped growth cones exhibited swaying of the C domain that terminates at membrane ruffles—also referred to as intrapodia—in the T zone [42] and a gradual decrease in the extent of membrane ruffles in the T zone over time. In contrast to the large growth cones, small, motile growth cones advanced by protruding lamellipodia between newly formed filopodia, while avoiding the formation of a large fan-shaped P domain (Figure 4(a), small growth cone on the right; Figure 4(b); Video 2). Furthermore, in smaller growth cones, the C domain followed more consistently the base of filopodia and maintained its directionality better than in large growth cones (Figure 4(b); Video 2). We measured the angle *α* between the mean orientation of the C domain boundary and the direction of growth cone advancement as an indicator for the persistency of the C domain advancement and engorgement (Figure 4(c)). We found that large, pausing growth cones frequently showed swaying of the mean C domain orientation along with splitting of the C domain, suggesting that persistent C domain advance could be more challenging for large growth cones (Figure 4(c)). On the other hand, small advancing growth cones exhibited a more persistent orientation of the C domain. After spending a significant amount of time (typically more than 3 h) in the pausing state, a large growth cone either completely retracted or retracted some of its P domain to form smaller growth cones. In this way, large growth cones gave rise to new neurites through branching from their original P domains (Figure 4(d)). In summary, the persistent advance of the C domain is more common in small growth cones and may contribute to the faster growth of small growth cones when compared to large fan-shaped growth cones.

### 3.5. The Cessation of Growth Cone Motility Correlates with Formation of Flat P Domain and T Zone

**Figure 5:**
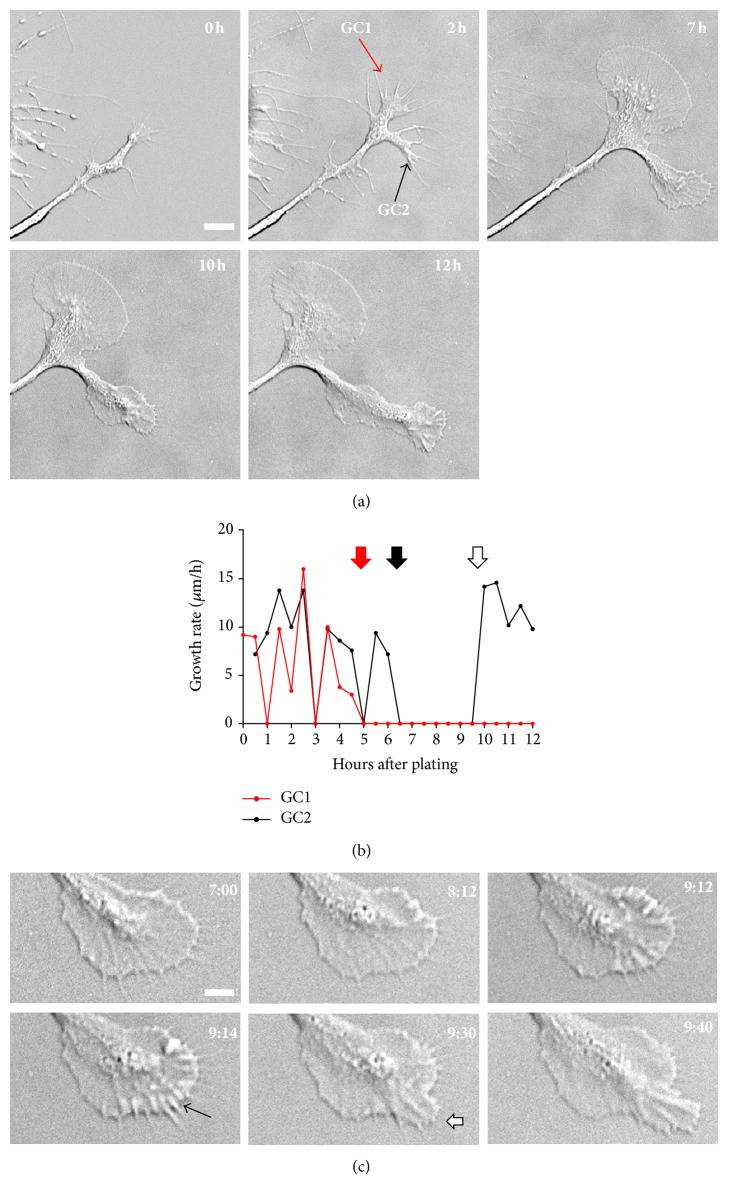
A flat P domain and T zone are indicative of immotile behavior. (a) DIC time-lapse series of two growth cones imaged for 12 h immediately after cell plating. Both growth cone 1 (GC1) and growth cone 2 (GC2) lost motility after generating flat P domain and T zone, whereas GC2 regained motility at 10 h after a three-hour pausing phase. (b) Plot of growth rate of GC1 and GC2, calculated at 30 min interval. Red and black arrows indicate the formation of flat P domain for GC1 and GC2, respectively. White arrow indicates when GC2 resumed protrusion of the P domain after pausing. (c) Time-lapse series of GC2 between 7 h and 9 h 40 min demonstrating the initial pausing followed by the recovery of the motile protruding state. Note the formation of membrane ruffles along the leading edge, which precedes P domain protrusion (black arrow at the 9 h 14 min time point). White arrow at 9 h 30 min time point indicates protrusion of filopodia and lamellipodia. Scale bar in (a): 10 *μ*m; scale bar in (c): 20 *μ*m.

Next, we went on to characterize the cellular events that lead to the formation of large growth cones, which is shown in Figure 5(a). In this example, two growth cones (GC1 and GC2, indicated at the 2 h time point) formed within 2 h of cell plating from a proximal neurite that remained following dissection of the ganglion (Figure 5(a); time point 0 h is immediately after cell plating; Video 3). The nascent growth cones advanced both by protrusion of filopodia and by “veillike” lamellipodia in-between filopodia. The growth rate fluctuated and was as high as 15 *μ*m/h. The expansion of lamellipodial veils eventually connected all filopodia, which formed a uniform array of spikes in the fan-shaped P domain of large growth cone 1 (GC1) at the 7 h time point. The flattening of both P domain and T zone coincided with the loss of growth cone motility in both large GC1 and small GC2 (red and black arrow in Figure 5(b)). By 7 h after plating, both growth cones reached the pausing state and resembled the larger growth cone shown in Figure 4(a). Whereas large GC1 never exited this pausing state and became completely retracted within the next 10 h (data not shown), smaller GC2 started to protrude again after formation of membrane ruffles both along the leading edge and in the T zone around 9 h and by elongation of filopodia from the P domain edge between 9 and 10 h (Figure 5(c); Video 3). Thus, the formation of flat P domain and T zone seems to be a hallmark of large, pausing growth cones. Furthermore, these observations suggest that local changes in actin and membrane dynamics need to occur in order to exit this pausing state.

### 3.6. Collagen I-Based Gel Promotes Faster Outgrowth of Bag Cell Neuronal Growth Cones

**Figure 6:**
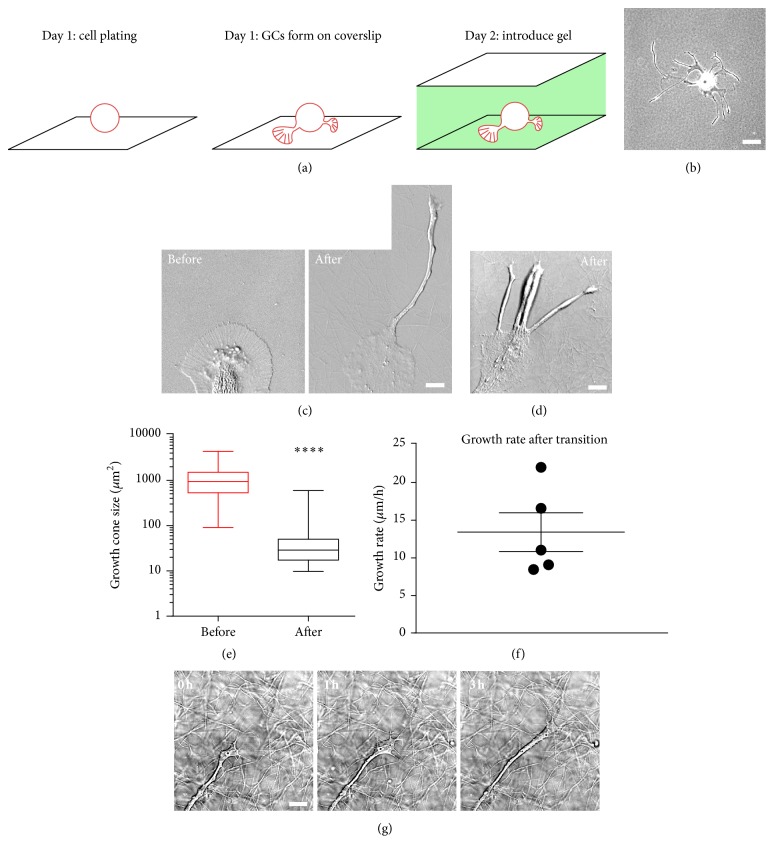
Collagen I gel rescues the motile behavior of bag cell neuronal growth cones. (a) Schematic depicting the time frame of the 2D-3D culture system. (b) Low magnification phase contrast image of a bag cell neuron cultured for 30 h in 3D culture system. Small growth cones appeared at the ends of long neurites. (c) High magnification DIC images of a growth cone before and after addition of the collagen gel. A rounded neurite developed from the leading edge of original P domain within 24 h. (d) Another example of a single growth cone that generated multiple neurites after introduction of the 3D culture system. (e) Statistical analysis of growth cone size before and after gel application. Average growth cone size ± SEM before gel application is 1238 ± 177.9 *μ*m^2^ (*n* = 37); average growth cone size after gel application is 70.3 ± 24.2 *μ*m^2^ (*n* = 25). Box: 25th and 75th percentile; whisker: min and max. ^*∗∗∗∗*^ *P* < 0.0001. Mann-Whitney test. (f) Growth rate of neurites after transition, measured from time lapse of individual growth cones as in (g). Average growth rate is 13.4 ± 2.6 *μ*m/h calculated from 5 growth cones in three independent experiments. (g) Time-lapse series of a growth cone advancing at the interface of 2D and 3D culture. Compared with the large growth cones in 2D cultures, this growth cone has a more compact morphology and advances at higher growth rates (10–20 *μ*m/h). Scale bar in (b): 60 *μ*m; scale bar in (c), (d), and (g): 10 *μ*m.

Our results so far suggested that the formation of large, fan-shaped growth cones after 1 day in culture was caused by a significant expansion of filopodia and lamellipodia, which are promoted by the two-dimensional (2D) PLL substrate. In an attempt to rescue large growth cones from this pausing state, we provided them with an additional substrate support from the dorsal side. In doing so, we designed an anisotropic three-dimensional (3D) collagen culture system. After growth cones had formed on PLL-coated coverslips, a layer of a collagen I-based gel containing 25%* Aplysia* hemolymph [40] and 20 *μ*g/mL PLL was introduced on top of the neurons, and the cells were cultured for an additional 24 h (Figure 6(a)). This 2D-3D transition in culture environment had a clear impact on the morphology of growth cones and promoted neurite outgrowth when compared to growth on 2D PLL substrates (Figure 6(b)).  Figure 6(c) shows a large growth cone on PLL before and after introduction of the collagen gel. Compared with the growth cone on plain PLL substrates, growth cones in 3D culture were consistently smaller and had less distinct domain separation (Figures 6(c) and 6(d)). Average total growth cone size before gel application was 1238 ± 178 *μ*m^2^ (*n* = 37) and after gel application was 70 ± 24 *μ*m^2^ (*n* = 25; Figure 6(e)). Time-lapse imaging of growth cones dynamics advancing at the 2D-3D interface revealed mean growth rates of 13.4 *μ*m/h (Figures 6(f) and 6(g); Video 4). Addition of hemolymph to the collagen gel was critical to convert the large pausing growth cones to smaller advancing growth cones. However, we do not believe that the changes observed were solely due to hemolymph, since growth cones cultured on planar surfaces coated with PLL and hemolymph result in a morphological phenotype that is midway between growth cones grown on PLL and at the 2D-3D interface [40]. The neurites formed after gel addition were more rounded and smooth, a sign of neurite consolidation (Figures 6(d) and 6(g); Video 4). These rounded neurites induced by the addition of 3D collagen gel were more similar to the preexisting neurite following dissection of the abdominal ganglion (Figure 5(a)) and different from the more spread neurites generated on the plain PLL substrates (Figure 4). Therefore, application of the collagen gel promoted neurite outgrowth of bag cell neurons, possibly by providing substrate support on the dorsal side of the growth cone and triggering smaller growth cones.

## 4. Discussion

### 4.1. Stages of Neurite Outgrowth of Cultured* Aplysia* Bag Cell Neurons

Growth cones formed by* Aplysia* bag cell neuron cultured on PLL-coated solid surfaces have proven to be an excellent model system for the past 30 years, as it enabled seminal discoveries regarding the nature of F-actin retrograde flow [23, 35], substrate-cytoskeleton coupling [24, 32, 43], microtubule and actin dynamics [27, 28, 33, 34], and cellular mechanotransduction [30–32]. Common features of the growth cones analyzed in the aforementioned studies are large size (typically with P domains larger than 100 *μ*m^2^), fan-shape, and clear domain distinction, the combination of which provides unique advantages especially for high-resolution measurements of cytoskeletal dynamics and cellular mechanics. While being a well-defined system, these large growth cones represent only one type among the diverse growth cone morphologies observed* in vitro* and* in vivo*. It is therefore necessary to investigate the motile behavior of bag cell growth cones beyond the typically reported shape to better compare findings made with different growth cone model systems. We believe that the basic mechanisms of how cytoskeletal dynamics is harnessed for growth cone motility and advance are highly conserved across different species and growth conditions. What varies among different growth cone systems, growth states, and conditions is the relative contribution of individual processes to net outgrowth, such as (1) actin assembly and recycling, (2) myosin activity, (3) adhesion and clutching, and (4) membrane delivery and recycling. Thus, the large, fan-shape* Aplysia* growth cone can be considered a transient “pausing growth cone,” in which actin assembly and retrograde flow rates are well balanced [27]. It can exit this pausing stage through a new stimulus such as an adhesion protein-coated bead or needle [24, 30], by branching or introduction of a collagen gel (this study) as further discussed below.

To achieve a more comprehensive assessment of the motile behavior of cultured bag cell growth cones, we analyzed the morphology and motility of bag cell growth cones on PLL-coated coverslip at different time points in culture in an unbiased manner with respect to growth cone morphology and size and describe how the large growth cones develop over time. During the first 24 h after plating, multiple large, fan-shaped growth cones formed with P domains larger than 100 *μ*m^2^. These growth cones formed from preexisting neurites or* de novo* from the cell body. The large P domain was induced by multiple lamellipodial veils protruding and connecting an increasing number of filopodia (Figure 5(a); Video 3). The formation of these large growth cones took about 3–5 h, and they typically spent a significant amount of time (more than 3 h) in the pausing state (Figure 4(a); Video 1). Large growth cones then typically branched into neurites with growth cones of smaller size between 24 and 72 h after plating (Figures 1, 3, and 4(d)). None of the neurites was morphologically distinct from others indicating that an axon cannot be distinguished from dendrites in bag cell neurites, which is not surprising considering that these cells primarily have neurosecretory function. Although a simple linear correlation between P domain size and growth rate for individual growth cones could not be established for all observed growth cones, a lower growth rate (smaller than 2 *μ*m/h) was usually found for large growth cones (P domain size more than 100 *μ*m^2^), which is in agreement with previous study where instantaneous growth rates of large growth cones were measured (Figure 2(b)) [40]. Small growth cones can advance as fast as 10 *μ*m/h on PLL substrates in the absence of any additional growth promoting factors (Figure 2(a); Video 2), suggesting the lower growth rate seen for large growth cones is likely due to the significant growth cone enlargement. When neurite outgrowth was promoted by either adding of hemolymph or plating on coverslips coated with hemolymph/laminin, a reduction in growth cone size could be readily observed [40, 44]. Bag cell neurons plated on laminin/hemolymph-coated silicone gel also displayed smaller size and faster growth rate [31]. Thus, a smaller size seems to be a prerequisite for fast growth in the case of* Aplysia* bag cell neuronal growth cones, whereas the large growth cones conventionally used for imaging cytoskeletal dynamics are representative of a pausing state. Because of this, one should therefore be careful when directly comparing absolute numbers such as retrograde flow rates gained from pausing* Aplysia* bag cell neuronal growth cones with faster-growing growth cones derived from other species or when extrapolating to growth cones* in vivo*. It will be interesting to investigate whether actin assembly and retrograde flow rates are different between large, pausing and small, advancing growth cones, in order to better understand the causal relationship between growth cone size and advance rate. A definite causal relationship between size and growth rate, however, could only be established if we would find a substrate-independent way to directly enlarge the size of fast moving* Aplysia* bag cell neuron growth cones.

### 4.2. Large and Small Growth Cone Have Different C Domain Dynamics and Ruffling Activity

Whereas the dynamics of large* Aplysia* bag cell growth cone has been extensively studied on shorter time scales, we have complemented this knowledge here with live cell imaging on longer time scales. Two cellular features were discovered when large growth cones developed from small growth cones: (1) the C domain frequently changed its direction when large growth cones were in a pausing state and sometimes split into two domains; (2) T zone ruffles appeared soon after the formation of a large growth cone and then gradually disappeared (Figure 4(a); Videos 1 and 3). Efficient engorgement of the C domain into the area where the P domain was previously located is an important step during the formation of axons [14]. Swaying of the C domain as observed in large growth cones (Figure 4; Video 1) may indicate a decision-making phase before final engorgement occurs in a particular direction. The varying angle between C domain orientation and the direction of growth in large growth cones (Figure 4(c)) could be caused by the symmetrically expanding P domain and the fact that there are not enough microtubules and vesicles to fill the expanding C domain evenly. When the symmetry in the P domain was broken by disruption of actin bundles, large growth cones could be reoriented [45]. Splitting of the C domain was usually followed by branching of growth cones. It appears that large growth cones can only exit the pausing state by branching into smaller ones, or they completely retract. Branching happened either spontaneously (Figure 4) or by applying a collagen gel (Figure 6(d)). A similar branching pattern was observed after treatment of Helisoma growth cones with conditioning factors [46]. In contrast, in fast-growing small growth cones, the P domain was biased towards the direction of a small number of filopodia, and C domain maintained better alignment with the direction of growth (Figures 4(b) and 4(c); Video 2). Interestingly, in small growth cones, ruffles in the P domain were often generated towards the direction of advancement along the leading edge, preceding the formation of filopodia from P domain (Figures 4(b) and 5(c); Video 3), whereas, in large growth cones, ruffles were usually confined to the T zone and eventually disappeared (Figure 4(a)). Whether the ruffles along the leading edge of small growth cones and in the T zone of large growth cones have the same function with respect to motility is unclear at this point. We speculate that the initial ruffling activity in the T zone of large growth cones may indicate an attempt of the growth cone to grow vertically as opposed to expanding horizontally.

### 4.3. Rescue of Growth Cone Translocation through 3D Collagen Gel

*In vivo* data suggests a correlation between growth cone morphology and motility: rapidly growing retinal ganglion cell growth cones in the optic nerve are smaller and have simple shape, whereas, at decision-making positions such as the optic chiasm, growth cones adopt a more elaborate fan-shape with filopodial and lamellipodial extensions and grow at a significantly reduced rate [7, 8]. The large growth cones from* Aplysia* bag cell neurons cultured on PLL may behave similarly to the second group, searching for chemical and physical cues that are scarce under the current culture conditions. For* Aplysia* bag cell neuron, this pausing state usually takes a long time (typically longer than 3 h) and redirecting its growth has been difficult. Physical interaction with either an* Aplysia* cell adhesion molecule- (apCAM-) coated bead or microneedle could lead to reorientation of C domain and increased advance rate [24, 30]. However, since the microneedle was kept in the same position in these experiments, increased neurite growth rate did not persist once the C domain reached the microneedle. Through application of collagen I-based gel containing hemolymph on top of the growth cones, we were able to create and maintain a topographical cue that could reliably rescue the forward translocation of large growth cones (Figure 6; Video 4). Growth cones found in this culture environment had smaller size, faster growth rate, and a dramatically different morphology that was reminiscent of growth cone on patterned substrate [47]. The neurons in this 2D-3D transition setup are not completely independent from the planar PLL-surface as indicated by the fact that neurites tend to remain close to the cover glass surface. To our knowledge, this 3D collagen gel is the only culture method that can induce well-consolidated neurites from* Aplysia* bag cell neurons. We believe the growth cones formed in this 2D-3D transition setup may be more similar to the growth cones in living animals with respect to morphology and motility. The relative contributions of individual processes of cytoskeletal and membrane dynamics may differ between the 2D and 3D environments resulting in the observed differences in morphology and motility. The effect of this collagen gel-based approach may be attributable to the presence of micron scale topographical cue that can be sensed and responded to by the growth cone. By creating and maintaining this local difference, individual growth cones maintain a rapidly advancing state similar to retinal ganglion cell growth cones in the optical tract. Although technically challenging, it will be interesting to quantitatively analyze motility, cytoskeletal dynamics, and force production of bag cell growth cones in 3D and compare with existing 2D data to better understand the machinery that powers growth cone advancement* in vivo*.

## 5. Conclusion

Here, we have shown that the typically observed large fan-shaped* Aplysia* bag cell growth cones formed on PLL-coated coverslip represent a specific pausing state early during cell culture, which is caused by a significant expansion of the lamellipodia and filopodia in the P domain. Small growth cones on PLL often grow faster than large growth cones, although a simple correlation between size and advance rate could not be established for small growth cones on PLL. An inverse correlation between growth cone size and growth rate, however, was found for large growth cones. The large pausing growth cones exhibited poor alignment between C domain and the direction of advancement, which could be indicative of the growth cone sampling the environment. In contrast, the small rapidly growing growth cones generated ruffles at the distal edge of P domain and grew as fast as 10 *μ*m/h without additional growth promoting factors. Application of collagen I-based gel promoted faster growth of bag cell growth cones and converted them into a more* in vivo*-like morphology. We conclude that significant lateral expansion of lamellipodia and filopodia as observed on PLL substrates during the initial culture period negatively affects neurite growth.

## Supplementary Material

Video 1. DIC time-lapse imaging of Aplysia growth cones on PLL-coated coverslip 48 h after plating. C domain in the large growth cone (left) swayed and split, while the P domain remained largely stationary except for small scale protrusions and retractions. In contrast, the small growth cone (right) advanced at the same time. Scale bar and time as indicated. Time compression: 1200x. Data for Figure 4a was taken from this video.
Video 2. DIC time-lapse imaging of Aplysia growth cone on PLL-coated coverslip 53 h after plating. A smaller growth cone showed persistent advancement of about 20 *μ*m in 3 h. Scale bar and time as indicated. Time compression: 1200x. Data for Figure 4b was taken from this video.
Video 3. DIC time-lapse imaging of two Aplysia growth cones on PLL-coated coverslip immediately after plating and followed for 12 h. The formation of extensive lamellipodia coincided with cessation of motility of left growth cone 1 (GC1 in Figure 5a). At 7 h, the stage was repositioned. The right growth cone 2 (GC2) resumed rapid advance at 9 h. Scale bar and time as indicated. Time compression: 5250x. Data for Figure 5a was taken from this video.
Video 4. DIC time-lapse imaging of Aplysia growth cone at the interface of PLL-coated coverslip and collagen I gel. Growth cone was compact and small, exhibited more filopodia than lamellipodia, and showed persistent advancement of about 20 *μ*m in 3 h. Scale bar and time as indicated. Time compression: 1200x. Data for Figure 6 was taken from this video.
